# Oral voriconazole monotherapy for fungal keratitis: efficacy, safety, and factors associated with outcomes

**DOI:** 10.3389/fmed.2023.1174264

**Published:** 2023-05-11

**Authors:** Youran Cai, Shimei Song, Yiying Chen, Xuyang Xu, Wenjin Zou

**Affiliations:** Department of Ophthalmology, The First Affiliated Hospital of Guangxi Medical University, Nanning, China

**Keywords:** fungal keratitis, voriconazole, treatment, efficacy, safety

## Abstract

**Purpose:**

To provide preliminary data on the efficacy and safety of oral voriconazole (VCZ) as a primary treatment for fungal keratitis (FK).

**Method:**

We performed a retrospective histopathological analysis of data on 90 patients with FK at The First Affiliated Hospital of Guangxi Medical University between September 2018 and February 2022. We recorded three outcomes: corneal epithelial healing, visual acuity (VA) improvement, and corneal perforation. Independent predictors were identified using univariate analysis, and multivariate logistic regression analysis was used to identify independent predictive factors associated with the three outcomes. The area under the curve was used to evaluate the predictive value of these factors.

**Results:**

Ninety patients were treated with VCZ tablets as the only antifungal drug. Overall, 71.1% (*n* = 64) of the patients had extreme corneal epithelial healing, 56.7% (*n* = 51) showed an improvement in VA, and 14.4% (*n* = 13) developed perforation during treatment. Non-cured patients were more likely to have large ulcers (≥5 × 5 mm^2^) and hypopyon.

**Conclusion:**

The results indicated that oral VCZ monotherapy was successful in the patients with FK in our study. Patients with ulcers larger than 5 × 5 mm^2^ and hypopyon were less likely to respond to this treatment.

## Introduction

Fungal keratitis (FK) is a serious infection of the cornea that often causes blindness and vision loss (1). The main cause is corneal trauma due to vegetable matter, organic materials and animal products that occur during agricultural work in developing countries (2). Previous studies have shown that the most common organisms that cause FK are *Fusarium* and *Aspergillus* (molds) and *Candida* (yeast) (3). The incidence of FK is >30% in developing countries (4, 5) and between 6 and 20% in developed countries for all microbial keratitis cases (6, 7). Although several new treatments have been developed, there is little evidence to guide treatment because there is a lack of effective antifungal agents (8).

Topical natamycin (5%) is the standard medical therapy recommended for FK. While it has broad activity, it has a poor ability to penetrate the intact corneal epithelium (9). Systemic antifungal agents have previously been used as an adjunct to topical treatment for ulcers and are thought to involve up to 50% of the stromal depth (10). Voriconazole (VCZ) is a triazole antifungal agent that is administered orally and intravenously. In recent years, corneal stromal injections of antifungal drugs have achieved good clinical results in the treatment of FK (11). However, whether VCZ improves clinical efficacy remains controversial (12). There has been no clinical research on oral VCZ as the primary antifungal therapy for FK. Our study was conducted to identify the effectiveness and safety of oral VCZ as the primary treatment for FK and the predictive factors of this monotherapy.

## Materials and methods

### Study design and population

This single-center retrospective study adhered to the tenets of the Declaration of Helsinki. This study was approved by the Ethics Review Board of the First Affiliated Hospital of Guangxi Medical University (E-2022-066). All data were anonymized and collected retrospectively, and the requirement for written informed consent was therefore waived. Between September 2018 and February 2022, patients diagnosed with FK and aged ≥18 years were recruited from the Department of Ophthalmology, the First Affiliated Hospital of Guangxi Medical University. We excluded any patients with a mixed infection in the history or on examination (bacterial, viral or parasitic), contraindications for the medication (allergic or cannot tolerate), an willingness to undergo regular review, a history of having received any antifungal treatment previously, and had an impending or a full thickness corneal perforation at an initial consultation.

### Clinical assessment and treatment

All eligible patients underwent slit-lamp examination by two experienced cornea specialists. The depth of ulcer infiltration and location of the ulcer were categorized at the slit lamp by a single ophthalmologist. Anterior augmented photographs (Topcon slit lamp and camera; Topcon Corp. Tokyo, Japan) were acquired at each visit for all patients. Corneal scrapings were collected from all suspected patients with clinical signs and symptoms suggestive of FK. All specimens were subjected to bacterial smears, bacterial cultures (blood agar), fungal smears, fungal cultures (agar slant Sabouraud medium), and fungal fluorescence staining. We also performed *in vivo* confocal microscopy (HRT III/RCM Heidelberg Engineering, Germany) in the suspected patients to confirm the diagnosis.

All the patients were administered oral VCZ (Chengdu Huashen Group Corp., China) tablets once the infection was confirmed. VCZ was administered at a loading dose of 400 mg twice daily for 24 h. Subsequently, a maintenance dose of 200 mg twice daily was administered until 2 weeks after the ulcer had healed. VCZ therapy did not exclude the use of other non-antifungal concomitant treatments, such as antibiotic agents or artificial tears when considered necessary by the cornea specialists. Patients had weekly monitoring of electrolyte and liver function tests.

### Data collection

All data were recorded using a standard protocol, including the demographics, visual acuity (VA, logMAR), history of trauma, presence of systemic disease, and duration of symptoms at the initial diagnosis. The signs of corneal ulcers were also recorded, including the ulcer size, ulcer location, depth of ulcer infiltration, and depth of hypopyon. The ulcer size and hypopyon depth were measured using Photoshop software. The location of the ulcer was categorized as the central area where the ulcer was located only in the center of the cornea (6 mm × 6 mm). A safety assessment was performed to evaluate and record any adverse events or complications that appeared during VCZ administration, including visual disturbances, neurological disorders (mental disorders and nervous system abnormalities), skin disorders, abnormal liver function and electrolyte disturbances.

In this study, we examined three primary outcomes: corneal epithelial healing, VA improvement, and corneal perforation. All patients were followed up for a minimum of 6 months after the initial oral VCZ. During the follow-up period, we recorded corneal ulcer healing, the VA, and corneal perforation at any time.

### Statistical analysis

All statistical analyses were performed using the IBM Statistical Package for Social Sciences (SPSS) version 26.0 (IBM, NY, USA). Data are presented as mean ± standard deviations/medians (minimum–maximum) and frequency percentages, as appropriate. Univariate analyses for proportions were compared using the chi-square test, independent *t*-test, or exact test, as appropriate. The prediction accuracy was measured using the area under the curve (AUC). For all the analyses, two-sided *p*-values were calculated. Statistical significance was set at *p* < 0.05. All graphs were prepared using GraphPad Prism 9.0 (GraphPad Software).

## Results

Ninety patients (53 men and 37 women) with a mean age of 56.74 ± 10.95 years (range: 28–83 years) and a mean duration of symptoms of 20 days (range: 1–90 days) were enrolled in the study. All patients were treated unilaterally. The most prevalent risk factor identified in these patients was injury due to vegetable matter (*n* = 29). Moreover, definite risk factors were not identified in any of the 22 patients. The preoperative demographic and clinical characteristics of the 90 patients are shown in Table 1.

**Table 1:** Demographic and clinical characteristics in patients with fungal keratitis (Patients, *n* [%]).

Characteristics	
Age, years	56.74 ± 10.95
Gender	
Male	53 (58.9)
Female	37 (41.1)
Eye	
Right	49 (54.4)
Left	41 (45.6)
VA baseline (logMAR)	2.16 ± 0.64
Duration of symptoms, days	20.00 (1.00–90.00)
Trauma	44 (48.9)
Vegetables	29 (32.2)
Brick	5 (5.6)
Animals	3 (3.3)
Wood	1 (1.1)
Chemical material	4 (4.4)
Dust	2 (2.2)
Systemic disease*	9 (10.0)
Hypertension	6 (6.7)
Diabetes	3 (3.3)
Hyperlipidemia	1 (1.1)
Hyperthyroidism	1 (1.1)
Medication time, days	53.00 (14.00–186.00)

VA, vision acuity; logMAR, logarithm of the minimum angle resolution; mm, millimeter. *Two patients had both hypertension and diabetes.

The fungal culture tested positive in 54 patients, with Fusarium species being the most common microorganism isolated (*n* = 24), followed by Aspergillus species (Table 2). There were no significant differences in efficacy and adverse events among the culture-positive and culture-negative populations (*p* > 0.05). Re-infection was noted in two eyes, which tested positive for *Dematiaceous* spp.

**Table 2:** Causative organisms of fungi cultured results of corneal ulcers.

Organisms	*N* (%)	Cornea healing	VA improved	Perforation
*Fusarium species*	24 (26.7)	15	10	5
*Aspergillus flavus*	10 (11.1)	7	7	2
*Aspergillus fumigatus*	6 (6.7)	4	5	0
*Curvularia species*	2 (2.2)	1	0	0
*Purpureocillium secies.*	1 (1.1)	1	0	0
*Bipolaris secies.*	1 (1.1)	1	1	0
*Acremonium secies.*	1 (1.1)	1	1	0
*Candida species*	1 (1.1)	1	1	0
Unidentified dematiaceous fungus	8 (8.9)	6	4	1
*Microscopy positive (no growth)*	36 (40.0)	27	22	5

**Figure 1:**
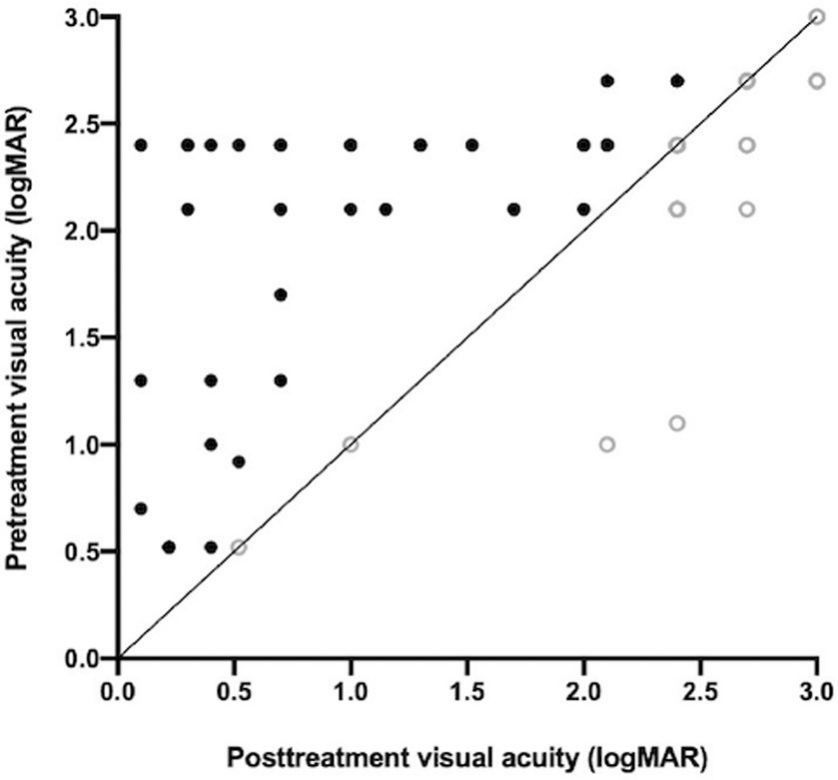
Scatter plot of pre-treatment and post-treatment visual acuity. Solid circles represent patients with improved vision, and hollow circles represent patients with unchanged or reduced vision after treatment.

All patients who had not used any antifungal drugs previously were administered oral VCZ tablets after the microbiological results of corneal scrapings or confocal microscopy results were obtained. The cure rate of corneal epithelial healing with oral VCZ as primary therapy was 71.1%. Vision improved in 51 (56.7%) patients and remained unchanged in 23 (25.6%) patients (Figure 1). The perforation rate throughout the treatment course was 14.4%. The results of the univariate analysis of the factors affecting the three outcomes are shown in Table 3. At three months, the VA baseline <2.40 (OR = 3.34, *p* < 0.05), the depth of ulcer infiltration <1/2CT (OR = 3.18, *p* < 0.05), the ulcer size <5 × 5 mm^2^ (OR = 4.07, *p* < 0.001) were more likely to achieve corneal epithelial healing. While patients with hypopyon were less likely to obtain corneal epithelial healing (OR = 0.10, *p* < 0.001). The difference in VA before and after treatment was statistically significant in all the patients (*p* < 0.001). injury due to vegetable matter and ulcer size were significant (*p* < 0.05). The depths of ulcer infiltration and hypopyon were nearly statistically significant (*p* = 0.056). Regarding corneal perforation, patients with corneal perforation showed significant differences in their age (*p* < 0.05). Perforation was negatively correlated to the depth of infiltration <1/2CT (OR = 0.12, *p* < 0.05) and ulcer size <5 × 5 mm^2^ (OR = 0.09, *p* < 0.001), and positively associated with hypopyon (OR = 11.44, *p* < 0.001). On multivariate logistic regression (Table 4), the ulcer size and hypopyon depth were independent predictors for 3-month corneal healing and corneal perforation (*p* < 0.05). For the 3-month VA improvement, only the depth of infiltration was an independent predictive factor. The number of patients with an ulcer size less than 5 × 5 mm^2^ was 4.17-fold higher than that with an ulcer size greater than 5 × 5 mm^2^ in terms of corneal healing and 0.14-fold lower in terms of perforation. The number of patients with a depth of corneal ulcer greater than 1/2CT was 3.54-fold higher than that with a depth less than 1/2CT in terms of an improvement in VA in three months.

**Table 3:** Univariate analyses for three outcome variables.

Predict factor	*n* = 90	3-month epithelial healing			3-month visual acuity improved			3-month cornea perforation		
		*n* (%)	Coefficient (95% CI)	*p*	*n* (%)	Coefficient (95% CI)	*p*	*n* (%)	Coefficient (95% CI)	*p*
Age		NA	NA	0.08^b^	NA	NA	0.06^b^	NA	NA	**0.044** ^**b**^
Gender			1.07 (0.43–2.70)	0.53^a^		0.82 (0.35–1.93)	0.67^a^		0.55 (0.17–1.79)	0.37^a^
Male	53	38 (71.7)			29 (54.7)			6 (11.3)		
Female	37	26 (70.3)			22 (59.5)			7 (18.9)		
VA baseline			3.34 (1.26–8.87)	**0.005** ^**a**^		0.48 (0.20–1.16)	0.10^a^		3.91 (0.81–18.86)	0.120^d^
<2.40	34	30 (88.2)			23 (67.6)			2 (5.9)		
≥2.40	56	34 (60.7)			28 (50.0)			11 (19.6)		
Duration of symptoms		NA	NA	0.25^c^	NA	NA	0.98^c^	NA	NA	0.62^c^
Systemic disease			0.78 (0.59–1.02)	0.44^d^		0.70 (0.47–1.04)	0.29^d^		0.84 (0.76–0.92)	0.35^d^
Yes	9	8 (88.9)			7 (77.8)			0 (0)		
No	81	56 (69.1)			44 (54.3)			13 (16.0)		
Trauma			1.17 (0.47–2.91)	0.82^a^		1.75 (0.75–4.07)	0.21^a^		0.88 (0.27–2.86)	0.83^a^
Yes	44	32 (72.7)			28 (63.6)			6 (13.6)		
No	46	32 (69.6)			2 (50.0)			7 (15.2)		
Injury with vegetable			1.42 (0.52–3.90)	0.62^a^		2.71 (1.04–7.06)	**0.038** ^a^		0.92 (0.26–3.29)	0.90^d^
Yes	29	22 (75.9)			21 (72.4)			4 (13.8)		
No	61	42 (68.9)			30 (49.2)			9 (14.8)		
Culture positive			0.73 (0.28–1.87)	0.64^a^		0.74 (0.31–1.74)	0.52^a^		0.94 (0.33–2.64)	0.90^a^
Yes	54	37 (68.5)			29 (53.7)			8 (14.8)		
No	36	27 (75.0)			22 (61.1)			5 (13.9)		
Central ulcer			1.75 (0.67–4.61)	0.26^a^		0.64 (0.27–1.49)	0.30^a^		0.40 (0.10–1.57)	0.18^a^
Yes	36	28 (77.8)			18 (50.0)			3 (8.3)		
No	54	36 (66.7)			33 (61.1)			10 (18.5)		
Depth of infiltrate			3.18 (1.20–8.44)	**0.008** ^a^		2.65 (1.32–5.31)	**0.001** ^a^		0.12 (0.02–0.95)	**0.027** ^a^
<1/2CT	33	29 (87.9)			26 (78.8)			1 (3.0)		
≥1/2CT	57	35 (61.4)			25 (43.9)			12 (21.1)		
Size of ulcer			4.07 (1.91–8.68)	**<0.001** ^a^		2.30 (0.97–5.46)	**0.056** ^a^		0.09 (0.02–0.43)	**0.001** ^a^
<5*5mm^2^	54	47 (87.0)			35 (64.8)			2 (3.7)		
≥5*5mm^2^	36	17 (47.2)			16 (44.4)			11 (30.6)		
Hypopyon			0.10 (0.03–0.29)	**<0.001** ^a^		0.43 (0.18–1.03)	**0.056** ^a^		11.44 (2.36–55.56)	**0.001** ^a^
Yes	36	16 (44.4)			16 (44.4)			11 (30.6)		
No	54	48 (88.9)			35 (64.8)			2 (3.7)		

Statistically significant values are bolded.

^a^Chi-Square test. ^b^Independent samples *t*-test. ^c^Mann–Whitney test. ^d^Fisher’s test. NA, Not Applicable; CT, Cornea Thickness.

**Table 4:** Multivariable analyses for three outcomes.

Predict factor	3-month corneal healing		3-month visual acuity		Perforation	
	Coefficient (95% CI)	*p*	Coefficient (95% CI)	*p*	Coefficient (95% CI)	*p*
Age					0.94 (0.87–1.02)	0.127
VA baseline	2.63 (0.68–10.10)	0.160				
Injury with vegetable			2.52 (0.90–7.09)	0.079		
Depth of infiltrate	1.79 (0.45–7.13)	0.410	3.54 (1.25–10.06)	**0.018**	0.49 (0.05–5.06)	0.553
Size of ulcer	4.17 (1.31–13.26)	**0.016**	1.55 (0.58–4.15)	0.380	0.14 (0.03–0.83)	**0.030**
Hypopyon	0.18 (0.55–0.58)	**0.004**	0.66 (0.25–1.76)	0.405	5.94 (1.09–32.47)	**0.040**

Statistically significant values are bolded.

**Figure 2:**
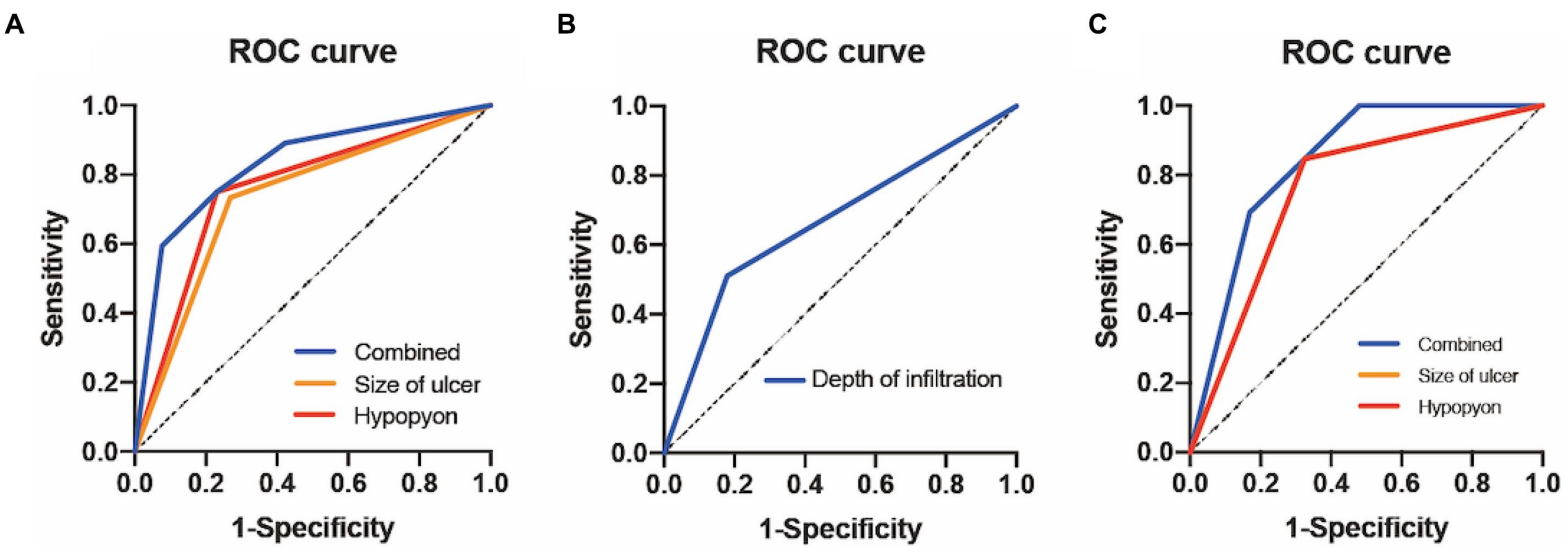
ROC curve analysis of FK for different outcomes. **(A)** ROC curve of 3-month corneal epithelial healing. **(B)** ROC curve of 3-month improved visual acuity. **(C)** ROC curve of 3-month corneal perforation. ROC, receiver-operating characteristic; FK, fungal keratitis.

The receiver-operating characteristic curve showed that the AUCs based on the ulcer size were 0.73 for predicting 3-month corneal healing and 0.76 for predicting corneal perforation. The AUCs based on the hypopyon depth were 0.76 for predicting 3-month corneal healing and 0.76 for predicting corneal perforation. The AUC based on ulcer infiltration was 0.67 for predicting 3-month VA improvement. In terms of combinations of two factors, the combined ulcer size and hypopyon depth were more accurate than only one factor for predicting both corneal healing (0.83) and perforation (0.84) (Figure 2).

Three patients healed spontaneously after perforation. We were able to improve perforation in five patients by performing conjunctival flap coverage, and the remaining patients underwent corneal transplantation. In two cases, the ulcers improved but did not heal, and we administered intrastromal injections of VCZ, which resulted in healing.

Overall, adverse events were reported in 7.8% of the patients (*n* = 7) during the study period. Four patients developed ocular hypertension, and all the patients responded to antiglaucoma medications. Liver function abnormalities were observed in one patient, which resolved two weeks after VCZ withdrawal. Two patients reported auditory or visual hallucinations, and none discontinued the medication because of adverse events. Symptoms in all the patients disappeared quickly after discontinuing treatment.

## Discussion

FK can present as superficial keratitis, corneal ulcers, or endothelial plaques. Fungal corneal ulcers are difficult to cure and contribute to blindness. Early detection and diagnosis of fungal corneal ulcers are significant. Currently, antifungal drug therapy remains the primary treatment option. VCZ has also been shown to have a broad spectrum and is the most promising treatment for FK (13). Additionally, oral VCZ may benefit patients who exhibit suboptimal responses to natamycin (14). Only a few cases have been reported on the effectiveness of using only oral VCZ for FK (15). To our knowledge, this is the first large case series to describe the effectiveness and safety in patients with FK using oral VCZ as a first-line antifungal drug.

VCZ can be administered topically (eye drops or injections) or systemically (oral tablets or injections). Topical VCZ eye drops have been used in many studies and have shown effective outcomes. However, eye drops should be produced on the day of treatment and remain stable during long-term storage (16). Oral VCZ has high bioavailability and can penetrate several parts of the eye (17). VCZ is a broad-spectrum antifungal agent that is effective against Candida, dematiaceous, and filamentous fungi (18). However, VCZ eye drops may be effective in most cases of FK but fail in cases involving Fusarium spp. (19). The Mycotic Ulcer Treatment Trial also revealed that natamycin is associated with better outcomes than those associated with voriconazole for the treatment of *Fusarium* keratitis (20). Our data suggest that oral VCZ monotherapy can cure 62.5% of cases caused by *Fusarium* spp., which is similar to the cure rate with *Aspergillus* spp. This may be affected by the small samples and low rate of culture positivity in our study. Voriconazole has been suggested as the first-line treatment for *Paecilomyces/Purpureocillium* keratitis (21). There was also one patient with *Purpureocillium* spp. in our study, in whom the cornea healed successfully, but the VA did not improve. Among culture-negative and positive patients, the corneal epithelial healing rate was 75.0 and 68.5%, respectively. This result is consistent with the previous studies about microbial keratitis (22, 23). However, studies regarding the comparison between culture-negative and positive fungal keratitis remain rare. Further investigations in this direction are required. The most dominant factor in our research was corneal trauma, which is consistent with results from China and other developing countries (24, 25). Oral VCZ is superior to other antifungal oral drugs, such as ketoconazole and fluconazole, providing a safe adjunct to topical therapy with good systemic absorption and high intraocular concentrations (26).

Similar to previous studies on FK (20, 27, 28), we used corneal epithelial healing as the primary outcome measure, with VA improvement and corneal perforation as the secondary outcome measures. We chose these because they provide the most objective and reproducible effect of therapy. Approximately 31% of patients with FK fail to respond to antifungal drugs, and some patients develop severe effects during the treatment (13). In our study, the overall cure rate of oral VCZ as the primary therapy was 77.1%. Ramakrishnan et al. studied 26 cases and found that only 50% of patients responded to topical VCZ. They summarized that peripheral infiltrates and hypopyon are possible predictive factors of the outcomes of topical VCZ treatment (29). Ting et al. studied patients with FK in the UK over a 10-year period and found that age > 50 years, VA <1.0logMAR, and an infiltration depth > 3 mm result in poor corneal epithelial healing (30). Chow et al. studied the visual prognosis of 103 patients with FK and found that a large ulcer size (> 4 mm), fungal ulcers in the central area, the presence of pus, and high intraocular pressure (> 21 mmHg) at the time of presentation are predictors of poor final visual outcomes (31). Our study demonstrated the statistical significance of systemic VCZ with a larger case series compared with previous research. Hung et al. reported that the presenting time, poor VA at the initial presentation, and trauma are significantly associated with VA recurrence (32). This is in contrast to our observations. We also suggest that hypopyon in the anterior chamber is an important independent predictor of corneal healing and perforation. However, this factor has rarely been mentioned in the literature on FK. Hypopyon may be a key factor not only in monotherapy but also in other antifungal therapies. Our data confirm that patients with hypopyon are less likely to exhibit corneal healing and non-perforation. To predict VA improvement, patients with ulcer infiltration of less than 1/2 CT will be more likely to approach VA improvement. A previous study revealed that the combination of oral VCZ and topical natamycin can cure 80% of patients with severe FK (10). The results of our analysis showed a significant difference in the size of the corneal ulcer and hypopyon depth. This demonstrates that using only oral VCZ as an antiulcer agent may be effective in patients with early and mild FK ulcers. Therefore, therapeutic options for patients with moderate-to-severe signs remain limited. To evaluate the factors for predicting the outcomes of VCZ monotherapy, we pursued the approach of combining two factors. The predictive value of these factors can be further enhanced by combining more than one predictor. Further research is required to assess the predictive value of other parameters associated with FK for other causes of curing, as these could further improve the prediction.

In addition to its therapeutic effects, we should also focus on the safety profile and side effects of oral VCZ medication. Hepatotoxicity, visual disturbances, photosensitivity, and skin rashes have been reported (33). In our study, an uncomfortable appearance was observed in 7.8% of the patients, including ocular hypertension, liver function abnormality, and hallucinations. After appropriate drug treatment, all the patients recovered after two weeks of drug distribution. Therefore, oral VCZ treatment usually requires hospitalization, placement in a quiet room, and observation for the development of complications. Since fungal keratitis occurs mostly in developing countries, some regions inevitably face difficulties in obtaining topical antifungal drugs promptly. Our study provides an alternative treatment choice to traditional topical antifungal therapy. Further studies comparing the method of oral antifungal tablets with conventional topical antifungal eyedrops are needed to fully evaluate its efficacy.

The sample size in our study was small, the patients were all from a developing country, and the pathogenesis was mainly agricultural trauma. Therefore, a larger sample size and diverse populations from different countries are needed. Our low positive fungal culture rate cannot exclude the possibility of false negatives. This resulted in our findings being unclear about which organisms are more suitable for this treatment. Additionally, there were some insufficiencies in the measurement approaches. The infiltration of the ulcer was judged subjectively by a specialist without objective measurements. Further investigations are required to confirm these factors and to gain a better understanding of possible factors.

## Conclusion

In summary, the results of the present study may provide a new treatment option for patients with FK. We confirmed that oral VCZ monotherapy is an effective treatment for fungal corneal ulcers and can induce good-quality remission with acceptable toxicity in most patients. We should consider a combination of these three factors, including the size of the ulcer greater than 5 × 5 mm^2^, infiltration of the ulcer, and presence of hypopyon, while discussing the treatment modality with patients. We recommend considering hospitalization for patients to take prompt action to manage side effects. Further studies on predictors of different antifungal medications in diverse populations should be conducted. This will help provide more standards for choosing drugs for patients with FK under various conditions.

## Data availability statement

The raw data supporting the conclusions of this article will be made available by the authors, without undue reservation.

## Ethics statement

This study was approved by the Ethics Review Board of the First Affiliated Hospital of Guangxi Medical University (E-2022-066). All data were anonymized and collected retrospectively, and the requirement for written informed consent was therefore waived.

## Author contributions

YRC designed the study and drafted the initial manuscript. SMS and YYC collected information and analyzed the data. XYX collated and interpreted the data. WJZ critically revised the manuscript. All authors have read and approved the final manuscript.

## Funding

This study was supported by the National Natural Science Foundation of China (81160119) and the Key Research and Development Program in Guangxi (AB20238003).

## Conflict of interest

The authors declare that the research was conducted in the absence of any commercial or financial relationships that could be construed as a potential conflict of interest.

## Publisher’s note

All claims expressed in this article are solely those of the authors and do not necessarily represent those of their affiliated organizations, or those of the publisher, the editors and the reviewers. Any product that may be evaluated in this article, or claim that may be made by its manufacturer, is not guaranteed or endorsed by the publisher.
